# Association of PNC, BC, and PM_2.5_ Measured at a Central Monitoring Site with Blood Pressure in a Predominantly Near Highway Population

**DOI:** 10.3390/ijerph120302765

**Published:** 2015-03-03

**Authors:** Mei Chung, Ding Ding Wang, Amanda M. Rizzo, Darrel Gachette, Marie Delnord, Ron Parambi, Choong-Min Kang, Doug Brugge

**Affiliations:** 1 The Department of Public Health and Community Medicine, Tufts University School of Medicine, Boston, MA 02111, USA; E-Mails: Mei_Chun.Chung@tufts.edu (M.C.); deenawang@gmail.com (D.D.W.); 2 Tufts University School of Arts, Sciences, and Engineering, College of Liberal Arts, Medford, MA 02145, USA; E-Mails: amanda.rizzo@tufts.edu (A.M.R.); darrelgachette@gmail.com (D.G.); 3 NSERM U1153, Obstetrical, Perinatal and Pediatric Epidemiology Research Team, Research Center for Epidemiology and Biostatistics Sorbonne Paris Cité (CRESS), Paris Descartes University, Paris 75270, France; E-Mail: mariedelnord@gmail.com; 4 Institute for Relevant Clinical Data Analytics (IRCDA), Boston Children’s Hospital, Boston, MA 02115, USA; E-Mail: dr.ronparambi@gmail.com; 5 Department of Environmental Health, Harvard School of Public Health, Boston, MA 02115, USA; E-Mail: cmkang@hsph.harvard.edu

**Keywords:** fine particulate matter, black carbon, ultrafine particles, blood pressure, acute

## Abstract

Elevated blood pressure is an indicator of cardiovascular stress and increased risk of cardiovascular-related morbidity and mortality. There is emerging evidence suggesting air pollutants, including particulate matter (PM), could promote hypertension, thereby increasing cardiovascular disease risk. Repeated measurement analyses were conducted to examine the associations of three types of PM with systolic blood pressure (SBP), diastolic blood pressure (DBP), and pulse pressure (PP) in 220 participants, (mean age = 58.5 years) from the Community Assessment of Freeway Exposure and Health study (CAFEH), most of whom live near a major highway. Ambient levels of air pollutants including particle number concentration (PNC; a measure of ultrafine particle (UFP) concentration), fine PM (PM_2.5_, Particle diameter <2.5 µm), and black carbon (BC) were measured at a central site <7 km from the study areas. Central sites are good at capturing short-term temporal trends in pollution associated with meteorological changes over regional areas. Linear mixed-effect models that accounted for repeated measures within one person were used to examine the associations between blood pressure variables and daily average of ambient PNC, PM_2.5_, or BC, controlling for demographic characteristics and major confounders including temperature. Our PNC model predicted that a higher PNC of 10,000 particles/cm^3^ was associated with higher DBP of 2.40 mmHg (*p* = 0.03), independent of other factors in the model. There were no significant associations for PM_2.5_ or BC. *Post hoc* subgroup analyses by obesity status showed that positive associations of DBP with PNC were more pronounced among obese individuals than non-obese individuals. These results suggested that PNC levels are associated with increased blood pressure, which may contribute to cardiovascular disease risk. More research is needed to assess the relationship between PNC and blood pressure and to address possible residual confounding.

## 1. Introduction

Particulate matter (PM) is a constituent of ambient air pollution [1]. Exposure to PM has been shown to produce adverse effects on cardiovascular (CV) health, including increased risk of morbidity and mortality from cardiovascular disease (CVD) [2,3,4,5]. Particles within the broad spectrum of PM emitted as byproducts of combustion range in size and chemical composition, and include PM less than 2.5 μm in diameter (PM_2.5_), black carbon (BC), and PM less than 100 nm in diameter (ultrafine particles, UFP, measured as particle number count, PNC) [1,5]. It is not certain which component(s) of PM are most responsible for observed deleterious CV effects.

Blood pressure (BP) elevation serves as an indicator of CV stress and increased risk of CV-related morbidity and mortality, as it suggests disruption of normative vascular homeostasis via complex pathophysiologic mechanisms [2]. There is a growing base of literature exploring the association between blood pressure and acute variability in ambient PM. Brook & Rajagopalan (2009) reviewed 11 epidemiologic and six controlled exposure studies that indicated positive correlations between PM and BP. Several other studies indicating an inverse or no relationship were also reported. While there is some heterogeneity in findings, the authors concluded that the evidence suggests that both short- and long-term exposure to PM_2.5_ is capable of producing pro-inflammatory vasoconstrictive events that can elevate BP [1]. This positive association between PM and BP is echoed across studies utilizing multiple cohort types and employing an array of monitoring methods.

More recently, Hoffman *et al.* (2012) found positive correlations between both PM_2.5_ and BC concentration (averaged from 1–5 days before examination) and BP in a cohort of subjects with type 2 diabetes mellitus [4]. Positive associations have also been observed in cohorts of cardiac rehabilitation patients, elderly subjects with coronary artery disease, schoolchildren, and young to middle-aged nonsmokers [3,5,6,7]. Ambient PM is most commonly measured by monitors located centrally to the study area. Assessing the association of central site pollution values with acute changes in biomarkers is a well-established approach. It has proven effective at discerning temporal (but not spatial) associations because pollutants, including those such as PNC that vary geographically, tend to rise and fall regionally based on common meteorology.

Protocols for BP measurements, however, are study-specific and variable. Delfino *et al.* (2010), for example, utilized ambulatory BP monitoring, whereas Hoffman *et al.* (2012) measured the brachial artery BP of the dominant arm [3,4]. It is also valuable to note that the association between systolic BP (SBP) and PM is better substantiated than for diastolic BP (DBP) and PM [5,6].

We examined the association of central site PNC, PM_2.5_, and BC with SBP, DBP and pulse pressure (PP). The study population was a subset of individuals participating in the Community Assessment of Freeway Exposure and Health study (CAFEH) [8]. PNC has been included in studies of association of BP in a few studies, with two finding positive associations [3,4,5]. To our knowledge, no prior studies have focused on populations that primarily reside near highways (where PNC is particularly elevated), as do most of the people enrolled in CAFEH.

## 2. Materials and Methods

### 2.1. Study Design and Recruitment

The study methods for CAFEH have been reported previously in detail [8]. Data from two near-highway areas and two paired urban background areas located in the City of Somerville and the Dorchester and South Boston neighborhoods of Boston, MA, were included in this analysis. Recruitment was for one year in each neighborhood and stratified for <100 m, 100–400 m, and >1000 m from the edge of Interstate 93 (I-93). Random samples were generated for all addresses within each area and every address in the random sample was approached. Inclusion of non-English speaking residents was improved by having complete sets of documents available in English, Spanish, Portuguese, Haitian Creole, Vietnamese, and Chinese, as well as field members fluent in each language. Recruitment was door-to-door by surveyors who were extensively trained and supervised. From the random sample, 174 provided clinical data. A convenience sample of clinical data from 94 participants was also recruited. The convenience samples mostly included residents in four elderly housing developments, two each in Somerville and Dorchester. The study protocol and consent forms were approved by the Tufts Health Sciences IRB (IRB#-10077), which is in compliance with the Declaration of Helsinki [9]. Participants provided signed consent forms for both the survey and separately for collection and storage of biological samples. The consent forms were retained with the participant’s data in confidential files.

### 2.2. Human Data

Enrolled participants completed a survey at their residence that included demographic information and smoking status, categorized as current, former, or never. Upon completing the in-home survey, participants were invited to attend two field clinics (the first typically within weeks of the home visit and the second months later) after fasting through the night. Clinics were held in the morning in the study areas near the highway. We assumed 9 a.m. for blood pressure measurement for the purposes of analysis because we did not have records of the exact time of each blood draw. We know that actual times varied between 7 a.m. and noon, with most between 8:00 a.m. and 10:30 a.m.

BP was taken with the participants seated using an automatic blood pressure machine, which minimizes influence of technique (Model #HEM711ACN2, Omron Healthcare, Kyoto, Japan). DBP and SBP were measured by a nurse in the right and then left arms (R-L). An additional BP measurement on the right arm was taken (measurement R-L-R) during second clinic visits in both study areas. Out of 204 participants in Somerville, 111 (54.4%) participants had three BP measurements (R-L-R); and in Dorchester, 93 out of 251 (37.0%). The average of R-L BP measures (termed average-arm SBP or DBP) was used as the dependent variable for our main analysis; and left-arm or right-arm BP measures were used for sensitivity analysis (described later in Statistical Methods). PP, the numeric difference between SBP and DBP, was calculated using the average R-L BP measures. A total of 270 participants attended a first clinic visit and 220 attended a second clinic, with BP measures at each clinic visit. Therefore, 50 participants had a BP measure at only clinic visit 1, while 220 participants had second BP measures. Two hundred and nineteen participants’ repeated blood pressure measurements were used for the models. Use of antihypertensive medications was recorded from medications at each home and coded by a physician. Height and weight were recorded using a standard scale (SECA, Model #8761321009) and stadiometer (Shorr Productions LLC, Model #905055).

As expected, left and right arm SBP and DBP measurements were strongly correlated with each other (*r* = 0.78 and 0.79, respectively, at the first clinic visit; and *r* = 0.80 and 0.65, respectively, at the second clinic visit). However, Bland–Altman limits-of-agreement plots [10] showed poorer agreement of between-arm SBP and DBP measurements at higher blood pressure (Figures S1 and S2). It should be noted that larger between-arm SBP and DBP measurements (≥10 mmHg) have been shown to be associated with higher risk of cardiovascular disease and mortality [11,12]. Thus, differences of between-arm SBP and DBP may be confounded by the underlying diseases of study participants.

### 2.3. Air Pollution and Temperature Data

Ambient PNC, PM_2.5_, and BC were measured on the roof (six floors above street level) of the Countway Library of Medicine at Harvard Medical School (HMS). The site is located on Huntington Avenue in Boston, fewer than 7 km from participant residences. Given the spatial variability of PNC, this distance likely introduced a degree of exposure misclassification. A Beta-Attenuation Mass Monitor (BAM, Met One Instruments Inc. Model 1020, Grants Pass, OR, USA) was used to measure hourly PM_2.5_ concentrations. The hourly concentrations were calibrated with the 24-h PM_2.5_ mass concentrations collected by the Harvard Impactor (HI). PNC was monitored continuously using a condensation particle counter (CPC, TSI Inc. Model 3022a, Shoreview, MN, USA). During the study period, the CPC malfunctioned from 1 March 2011 to 8 August 2011, which resulted in about seven months of missing PNC data. Our PNC analysis excludes blood pressure measurements during this time period. Black carbon (BC) was measured continuously using an Aethalometer (Magee Scientific Corp., Model AE-21, Berkeley, CA, USA) based on optical transmittance at a wavelength of 880 nm. We obtained temperature data from the weather station at Boston Logan airport.

The daily average of each pollutant and temperature were calculated using 24-h moving averages of the hourly data prior to 9 a.m. on each clinic visit date for each participant. Any missing hourly data within the 24 h resulted in missing daily averages.

### 2.4. Statistical Methods

The analysis presented here was for temporal variability only, consistent with similar studies in the literature that assess short-term variation in biological measures relative to central site air monitoring data. We used maximal likelihood mixed-effects, repeated-measures models to evaluate the associations between SBP or DBP and daily average of ambient PNC, PM_2.5_, or BC. Our models accounted for the correlations between the repeated measures within one person. Mixed models included a random intercept for each person and an unstructured (assumption free) covariate matrix structure.

### 2.5. Dependent, Independent Variables, and Covariates

For each model, the dependent variable was PP or the average of SBP or DBP in both arms. Sensitivity analyses using first right-arm, left-arm, and the repeated right-arm (second clinic only) SBP or DBP were conducted for all statistical models. Daily average (the 24 h prior to 9 a.m. on the clinic date) of PNC (number/cm^3^), PM_2.5_ (μg/m^3^), or BC (μg/m^3^) was the main independent variable. Both dependent and independent variables were time-varying. Daily average temperature (°C) was included in all models as a time-varying covariate. The quadratic term of the centering temperature measure was used in the PNC model because the untransformed temperature measure was highly collinear with PNC (*r* = −0.75). The correlation matrix of pollutants, temperature, and quadratic-centering transformed temperature measure is shown in Table S1. Because temperature is known to affect BP, the influence of temperature on the associations between each pollutant and BP was tested by removing the quadratic term of the centering temperature variable from our models. A change in beta coefficient for each pollutant of more than 10% was used to evaluate whether temperature is a confounder in the associations between each pollutant and PP. Other time-varying covariates in our models are seasonal variation (sine and cosine of calendar date), weekend or weekdays, and long-term temporal trends (calendar day as a linear continuous variable).

The same set of time-constant covariates, measured only at baseline, was adjusted in all models including: age (year), gender (female or male), race (white, Asian, black, or other), income level (<$25,000, $25,000–$74,999, ≥$75,000, or don’t know/refused), education level (<high school, high school, undergraduate, or graduate), smoking status (never, former, or current), obesity status (obese or non-obese), use of hypertension medications (yes or no), sampling method (random or convenience sample), and distance to highway I-93 (≤400 or ≥1000 m). Demographic covariates (*i.e.*, age, gender, race, income, and education levels) were chosen *a priori* and left in all models without considering their statistical significance. Other covariates were chosen based on prior knowledge of potential confounders for their associations with both blood pressure and traffic-related air pollution [4,13,14].

Multi-collinearity and model fitting statistics were checked when adding these covariates. An interaction term between obesity status and hypertension medications was tested in all models but dropped out because there was no statistically significant interaction. Tests for normality indicated that SBP at both visits and DBP at visit 2 were skewed. We repeated all analyses using natural log transformed BP as the dependent variables and found consistent results compared with our main analyses. We chose to present results using untransformed BP measures as our main results because their coefficients are more easily interpreted.

Most variables included in the regression models had 1% or less missing data. However, BMI and smoking status had 8% and 4% missing, respectively. Analyses were conducted using STATA SE 12.0 statistical software (Stata Corp., College Station, TX, USA). Robust standard errors were calculated using the Huber-White Sandwich Estimator. All *p* values were two tailed, and a *p* value less than 0.05 was considered to indicate statistical significance. Results are reported in mean ± standard deviation unless otherwise noted.

## 3. Results

**Figure 1:**
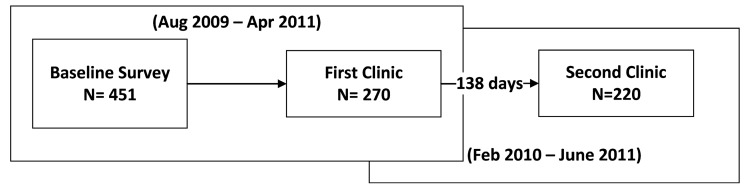
Flowchart of clinic visit 1 and 2.

During the study period from August 2009 to June 2011, 270 participants attended a first clinic visit and 220 attended a second clinic (Figure 1). The two clinic visits were an average of 138 (35–364) days apart. The demographic characteristics of the study participants who attended the first and/or second clinic visit were similar (Table 1). The majority of study participants were middle-aged (mean age 58.0 for clinic 1; 58.5 for clinic 2), white (66%; 68%), female (61%; 62%), and lived near a major highway (I-93) (80%; 81%). More than half were overweight (mean BMI 29.8; 29.6) and more than one-third were obese (36%). The participants’ blood pressure varied slightly between the two clinics (Table 2). On average, blood pressure was higher in the first clinic visit (average-arm SBP = 136 mmHg and DBP = 78 mmHg) than the second clinic visit (average-arm SBP = 131 mmHg and DBP = 75 mmHg). This may reflect seasonal variations in blood pressure because first clinic visit dates were mostly in winter while second clinic dates were mostly in summer.

**Table 1:** Demographic characteristics of CAFEH participants who participated in at least one clinic visit.

Demographic Characteristics	1st Clinic Visit (*n* = 270)	2nd Clinic Visit (*n* = 220)
Age (year)	58.0 (11.7)	58.5 (11.5)
Female	164 (61%)	135 (62%)
*Race*	
White	179 (66%)	149 (68%)
Asian	31 (11%)	25 (11%)
Black	16 (6%)	10 (5%)
Other	44 (16%)	36 (16%)
*Education*		
Less than high school	57 (21%)	40 (18%)
High school	83 (31%)	66 (30%)
Undergraduate	79 (29%)	69 (31%)
Graduate	51 (19%)	45 (20%)
*Income*		
Less than $24,999	30 (11%)	23 (10%)
$25,000–$74,999	99 (37%)	78 (35%)
$75,000 or more	87 (32%)	71 (32%)
Don’t know/ refused	54 (20%)	48 (22%)
Random sample	176 (65%)	140 (64%)
*Highway Proximity*		
<400 m	216 (80%)	175 (81%)
>1000 m	53 (20%)	40 (19%)
*Smoking Status*		
Never smoked	97 (37%)	84 (40%)
Used to smoke	102 (39%)	79 (37%)
Current smoking	63 (24%)	46 (22%)
Body Mass Index (kg/m^2^)	29.8 (7.3)	29.6 (6.6)
Obese	97 (36%)	80 (36%)
Hypertension	97 (36%)	82 (37%)

Note: Data in the table were expressed as mean (SD) or n (%).

**Table 2:** Systolic and diastolic blood pressure measurement during clinic visit 1 and visit 2. Data for visit 1 are presented for the full study population and restricted to those who had a second visit.

Blood Pressure	Visit 1	Visit 1 *	Visit 2
	*n* = 270	*n* = 220	*n* = 220
*Systolic*	*Mean (SD) or n (%)*		
Average (mmHg)	135.8 (19)	135.8 (19)	131.1 (19)
Left arm (mmHg)	132.8 (19)	132.5 (18)	129.0 (20)
Right arm (mmHg)	138.6 (21)	138.9 (21)	132.9 (20)
Arm diff > 10 mmHg *	105 (39%)	92 (42%)	79 (37%)
*Diastolic*				
Average (mmHg)	78.3 (11)	78.0 (10)	75.3 (11)
Left arm (mmHg)	77.9 (11)	77.4 (11)	75.0 (12)
Right arm (mmHg)	78.7 (12)	78.5 (11)	75.5 (13)
Arm diff > 10 mmHg **	38 (14%)	31 (14%)	43 (20%)

Notes: Visit 1 *: Restricted to those also attended Visit 2; Data are expressed as mean (SD) or n (%). Arm diff = BP difference between left and right arm within person. ** *n* = 216 for the difference in BP between arms.

Forty-two percent (42%) of the participants at first clinic visits and 36% at the second clinic had a SBP difference greater than 10 mmHg between right and left arms. The DBP difference between arms was less, with a 14% difference in first clinic and 20% in second clinic (also see Figures S1 and S2).

We looked at the correlation among the three pollutants before putting them in mixed models and found that PM_2.5_ and BC were closely correlated to each other (*r* = 0.79). PNC, on the other hand, was poorly correlated with the other two pollutants (*r* = −0.01 to PM_2.5_ and *r* = 0.30 to BC; Table S1).

Hourly levels of PNC, PM_2.5_, and BC were used to calculate 24-h means. Table 3 shows the 24-h mean of each air pollutant by clinic visit. BC and PM_2.5_ were similar between clinical visits 1 and 2, while PNC was lower during the warmer seasonal period of the second clinic visits, as expected based on extensive mobile monitoring for the study areas in which participants lived [15].

**Table 3:** Summary statistics of mean air pollutant concentrations in Boston, Massachusetts.

Air Pollutant	Visit 1				Visit 2			
	n	Mean (SD)	min	max	n	Mean (SD)	min	max
PNC (number/cm^3^) *	209	17,000 (5800)	4700	29,000	126 *	8300 (5100)	3900	27,000
PM_2.5_ (µg/m^3^)	270	7.30 (4.3)	0.78	20.4	222	7.80 (3.7)	2.38	20.9
BC (µg/m^3^)	261	0.68 (0.4)	0.25	1.62	203	0.62 (0.3)	0.18	1.59

Notes: * PNC values missing due to the CPC malfunctioning for part of clinic 2 (March 2011–August 2011).

### 3.1. PNC Model

The average-arm DBP model, but not the SBP or PP model, was found to have a significant association with PNC after controlling for demographics, temperature, seasonal variation, and other major confounders (*p* = 0.03; Table 4). Our PNC model predicted that with every 10,000 particles/cm^3^ of higher PNC, DBP was 2.40 mmHg higher, independent of other factors in the model. Using natural log transformed BP as the dependent variable, our PNC model predicted that for every 10,000 particles/cm^3^ that PNC was higher, DBP was 2.8% higher (*p* = 0.05) (Table S2). Other independent factors associated with higher average-arm DBP were Asian race (*p* = 0.001) and obesity (*p* = 0.001).

**Figure 2:**
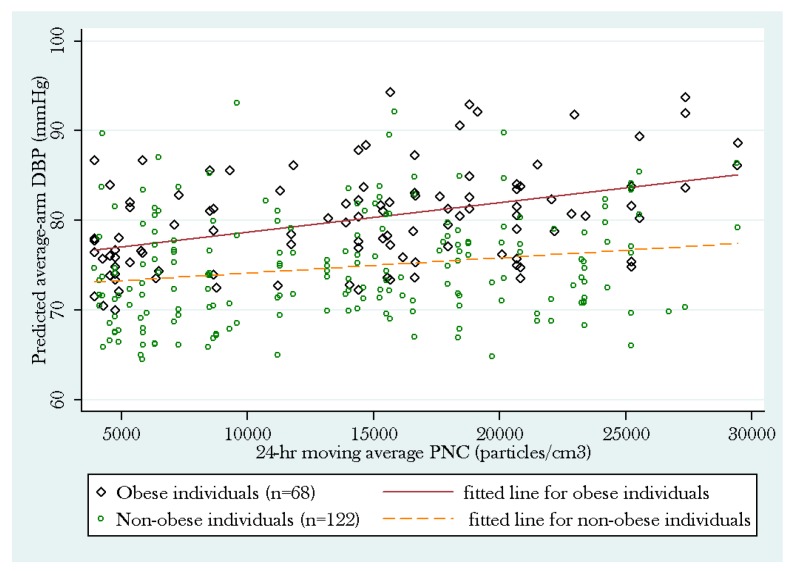
Subgroup analysis of the PNC model by obesity status.

*Post hoc* subgroup analyses by obesity status showed that the positive association between average-arm DBP and PNC was more pronounced among obese individuals (*p* = 0.043) than non-obese individuals (*p* = 0.53). The subgroup analysis among non-obese individuals did not reach statistical significance but showed a similar trend (Figure 2).

**Table 4:** Linear Mixed Model of PNC (number of observations = 302 in 190 people).

Dependent variable: SBP, DBP, or PPPNC Model	SBP			DBP				PP	
	R	Robust SE	P	R	Robust SE	P	R	Robust SE	P
Predictor: 24 h PNC (10,000 particles/cm^3^) ^†^	2.19	1.82	0.23	2.40	1.11	0.03 *	−0.16	1.34	0.91
Quad-center transformed measure—24 h avg temp (°C)	−0.01	0.01	0.43	−0.01	0.01	0.24	−0.001	0.01	0.88
Random *vs.* Convenience Sample	2.77	3.18	0.38	−0.04	1.96	0.98	2.89	2.17	0.18
Highway proximity (≤400 *vs.* ≥1000 m)	−1.49	2.65	0.57	0.98	1.86	0.60	−2.42	2.14	0.26
Obesity status (obese *vs.* non-obese)	7.07	2.43	0.00 **	5.91	1.63	0.00 **	1.32	1.84	0.47
Hypertension medication use (yes *vs.* no)	−1.94	2.78	0.48	−1.59	1.76	0.37	−0.56	2.09	0.79
Age (year)	0.86	0.11	0.00 **	0.03	0.07	0.69	0.83	0.09	0.00 **
Gender (female *vs.* male)	−3.57	2.46	0.15	−2.46	1.55	0.11	−1.22	1.74	0.49
*Race (White)*									
Asian	14.99	4.82	0.00 **	9.78	2.64	0.00 **	5.25	3.67	0.15
Black	9.06	6.49	0.16	5.17	3.82	0.18	3.68	3.29	0.26
Other	4.17	3.08	0.18	2.11	2.36	0.37	2.12	2.00	0.29
*Smoking status (Never smoked)*									
Used to smoke	1.41	2.48	0.57	1.27	1.75	0.47	0.21	1.84	0.91
Current smoker	−0.04	3.21	0.99	2.41	2.05	0.24	−2.45	2.36	0.30
*Education (<high school)*									
High school	−3.44	3.58	0.34	−4.81	2.50	0.05	1.35	2.58	0.60
Undergraduate	−4.99	3.64	0.17	−2.22	2.37	0.35	−2.69	2.64	0.31
Graduate	−10.06	3.83	0.01 *	−5.79	2.61	0.03	−4.24	2.63	0.11
*Clinic Dates*	**								
Seasonal Variation ^1^	−1.00	1.29	0.44	−0.32	0.67	0.63	−0.75	0.99	0.45
Seasonal Variation ^2^	1.93	1.26	0.13	−0.68	0.85	0.43	2.45	1.00	0.01 *
Weekdays *vs.* Weekend	−0.04	1.83	0.98	0.01	1.06	0.99	−0.18	1.42	0.90
Clinic Date (1 day)	−0.02	0.01	0.00 **	0.005	0.004	0.30	−0.02	0.01	0.00 **

**Notes: ^†^** PNC beta value was converted to reflect an increase per 10,000 particles per cubic centimeter. * *p* ≤ 0.05, ** *p* ≤ 0.01; ^1^ Seasonal variation using sine of clinic dates.^ 2^ Seasonal variation using cosine of clinic dates.

In contrast to the PNC models, we did not observe significant associations between average-arm SBP, DBP, or PP with PM_2.5_ or BC. The PM_2.5_ and BC models reacted similarly to the covariates including seasonal variations, long-term trends, age, obesity status, and race, except that lower temperature was significantly associated with higher SBP and DBP in both models (Table 5 & Table 6). By removing the quadratic term of the centering temperature variable from our models, the beta coefficients dramatically changed (much higher than 10%) for all PM_2.5_ models and for BC models. However, the changes in beta coefficients were less than 10% for PNC models, except for the association between PNC and PP (Table S3).

### 3.2. Sensitivity Analyses

The same models for each pollutant were repeated using first right-arm, left-arm, and the repeated right-arm BP measures as the dependent variables to see whether choice of BP measures would affect our findings. The sensitivity analyses resulted in similar associations compared with our main analyses using average-arm BP measures. Specifically, the sensitivity analysis models for the first right-arm BP measures (at both visits) predicted that if PNC was higher by 10,000 particles/cm^3^, DBP would be higher by 2.45 mmHg (*p* = 0.07). The models using the repeated right-arm BP measures (at visit 2 only) found significant associations with PNC higher by 10,000 particles/cm^3^ associated with a 4.05 mmHg higher SBP (*p* = 0.05) and a 2.33 mmHg higher DBP (*p* = 0.08). Using the left arm, the association between PNC and DBP was *p* = 0.06, or just outside statistical significance.

Sensitivity analyses using first right-arm, repeated right arm, or left-arm only BP as the dependent variables did not result in any statistically significant associations for models of PM_2.5_ and BC, although the association just missed statistical significance (*p* = 0.06) for repeated right arm PP with BC (Table S4).

## 4. Discussion

We found that central site PNC levels in the preceding day were significantly associated with higher levels of DBP. We did not find a statistically significant association between either BC or PM_2.5_ and BP in our main analyses. Our finding that PNC was poorly correlated with BC or PM_2.5_ is supported by the literature [3,4]. It also suggests that the associations of PNC with BP that we observed would be unlikely to be due to effects from exposure to either BC or PM_2.5_. However, it is at least possible that the effects (not the association) were related to BC or PM_2.5_ if the PNC measures were a better exposure estimate for BC and PM_2.5_ at the participant’s home. We found that temperature was a significant confounder in BC and PM_2.5_ models, but not in the PNC models after adjustment for other potential confounders including seasonal variations. Thus, the associations between PNC levels and BP that we observed wereunlikely to be confounded by temperature or seasonal variation in blood pressure.

**Table 5:** Linear Mixed Model of PM_2.5_ (number of observations = 436 in 243 people).

Dependent variable: SBP, DBP, or PPPM_2.5_ Model	SBP			DBP				PP	
	R	Robust SE	P	R	Robust SE	P	R	Robust SE	P
Predictor: 24 h PM_2.5_ (µg/m^3^)	−0.21	0.16	0.20	−0.14	0.11	0.21	−0.06	0.11	0.58
Quad-center transformed measure—24 h avg temp (°C)	−0.02	0.01	0.02 *	−0.01	0.01	0.08	−0.01	0.01	0.09
Random *vs.* Convenience Sample	4.43	2.39	0.06	0.02	1.45	0.99	4.50	1.82	0.01*
Highway proximity (≤400 *vs.* ≥1000 m)	−1.01	2.48	0.69	0.55	1.54	0.72	−1.58	2.30	0.49
Obesity status (obese *vs.* non-obese)	9.33	2.09	0.00 **	6.81	1.37	0.00 **	2.58	1.59	0.11
Hypertension medication use (yes *vs.* no)	−0.98	2.50	0.70	−1.61	1.50	0.28	0.62	1.85	0.74
Age (year)	0.73	0.10	0.00 **	−0.01	0.06	0.87	0.74	0.09	0.00 **
Gender (female *vs.* male)	−4.02	2.07	0.05	−1.98	1.23	0.11	−2.06	1.56	0.19
*Race (White)*									
Asian	11.16	4.31	0.01 *	8.56	2.35	0.00 **	2.70	3.39	0.43
Black	2.86	4.69	0.54	4.79	2.35	0.04 *	−2.00	3.27	0.54
Other	0.54	3.10	0.86	1.29	2.07	0.53	−0.79	2.26	0.73
*Smoking status (Never smoked)*									
Used to smoke	−2.20	2.28	0.34	−0.90	1.47	0.54	−1.33	1.74	0.45
Current smoker	−2.93	2.67	0.27	−0.86	1.61	0.59	−2.12	2.05	0.30
*Education* *(<high school)*									
High school	−3.01	3.21	0.35	−2.53	2.10	0.23	−0.53	2.40	0.83
Undergraduate	−5.09	3.28	0.12	−2.79	1.94	0.15	−2.38	2.49	0.34
Graduate	−9.28	3.48	0.01 *	−4.65	2.27	0.04 *	−4.74	2.50	0.06
*Clinic Dates*	**								
Seasonal Variation ^1^	0.14	0.95	0.89	0.89	0.51	0.08	−0.75	0.74	0.31
Seasonal Variation ^2^	1.79	0.93	0.05	0.45	0.62	0.47	1.27	0.68	0.06
Week day *vs.* Weekend	−1.32	1.58	0.40	−0.56	0.91	0.54	−0.96	1.24	0.44
Clinic Date (1 day)	−0.02	0.01	0.00 **	−0.01	0.00	0.12	−0.01	0.00	0.01 *

Notes: * *p* ≤ 0.05, ** *p* ≤ 0.01; ^1^ Seasonal variation using sine of clinic dates. ^2^ Seasonal variation using cosine of clinic dates.

**Table 6:** Linear Mixed Model of BC (number of observations = 436 in 243 people).

Dependent variable: SBP, DBP, or PPBC Model	SBP			DBP			PP		
	R	Robust SE	P	R	Robust SE	P	R	Robust SE	P
Predictor: 24 h BC (µg/m^3)^)	−1.33	2.43	0.58	−1.26	1.69	0.46	−0.03	1.76	0.99
Quad-center transformed measure—24 h avg temp (°C)	−0.02	0.01	0.01 *	−0.01	0.01	0.04 *	−0.01	0.01	0.06
Random *vs.* Convenience Sample	4.39	2.40	0.07	0.01	1.45	1.00	4.47	1.82	0.01 *
Highway proximity (≤400 *vs.* ≥1000 m)	−1.17	2.48	0.64	0.46	1.55	0.77	−1.64	2.30	0.48
Obesity status (obese *vs.* non-obese)	9.34	2.10	0.00 **	6.80	1.37	0.00 **	2.59	1.59	0.10
Hypertension medication use (yes *vs.* no)	−1.03	2.50	0.68	−1.66	1.51	0.27	0.61	1.85	0.74
Age (year)	0.74	0.10	0.00 **	−0.01	0.06	0.89	0.74	0.09	0.00 **
Gender (female *vs.* male)	−4.14	2.07	0.05	−2.04	1.23	0.10	−2.11	1.56	0.18
*Race (White)*									
Asian	11.15	4.33	0.01 *	8.54	2.36	0.00 **	2.71	3.40	0.43
Black	2.75	4.68	0.56	4.69	2.37	0.05	−2.01	3.25	0.54
Other	0.68	3.08	0.83	1.40	2.05	0.50	−0.76	2.25	0.74
*Smoking status (Never smoked)*									
Used to smoke	−2.21	2.28	0.33	−0.92	1.47	0.53	−1.32	1.74	0.45
Current smoker	−3.01	2.68	0.26	−0.92	1.62	0.57	−2.14	2.06	0.30
*Education* *(< high school)*									
High school	−2.96	3.21	0.36	−2.50	2.10	0.24	−0.51	2.39	0.83
Undergraduate	−4.97	3.28	0.13	−2.73	1.94	0.16	−2.33	2.49	0.35
Graduate	−9.21	3.48	0.01 *	−4.60	2.27	0.04 *	−4.72	2.49	0.06
*Clinic Dates*	**								
Seasonal Variation ^1^	0.10	0.96	0.92	0.87	0.51	0.09	−0.78	0.75	0.30
Seasonal Variation ^2^	1.95	0.94	0.04 *	0.58	0.63	0.35	1.31	0.70	0.06
Week day *vs.* Weekend	−1.24	1.57	0.43	−0.51	0.90	0.57	−0.94	1.24	0.45
Clinic Date (1 day)	−0.02	0.01	0.00 **	0.00	0.00	0.14	−0.01	0.00	0.01 *

Notes: ^1^ Seasonal variation using sine of clinic dates; ^2^ Seasonal variation using cosine of clinic dates; * *p* < 0.05; ** *p* < 0.01.

While there is evidence suggesting that exposure to PM_2.5_ may be associated with elevation of SBP and DBP, there are also a small number of studies with null findings similar to ours [1,2,3,4,5,6]. Brook and Rajagopalan (2009) acknowledged these inconsistencies, but ultimately concluded that PM_2.5_ elevation is capable of disrupting vascular hemodynamics to induce hypertension [1]. The literature also suggests that BC exposure may be associated with elevated BP [3,4], which we also did not see in our analyses.

Our finding that PNC in the preceding day was significantly associated with DBP elevation is consistent with two studies [3,5] and in contrast to one other that included PNC in its models [4]. A distinction in our study population was that most of the participants lived in close proximity to a highway, where PNC levels are known to be elevated [15]. We previously demonstrated a median Pearson correlation of 0.59 between the central site used here and a subset of homes in the CAFEH study area [16]. The correlation between near highway and central site PNC values is likely driven by common meteorology and traffic patterns at both sites. However, there is almost certainly exposure misclassification of unmeasured magnitude for our study participants relative to the central site PNC levels, albeit the temporal trends are likely similar in both locations. Together with possible residual confounding, this limits the confidence with which we can interpret our results.

Our findings are biologically plausible as there is mounting evidence in the literature that PM inhalation leads to a cascade of systemic inflammatory and oxidative stress responses, as well as stimulation of the autonomic nervous system, which is associated with increased cardiovascular risks [17,18,19]. Additionally, PM toxicity has been reported to vary based on particle size, composition, and source of exposure [20]. PM-toxicity may be mediated by both direct and indirect biological mechanisms. For example, due to their extremely small size, ultrafine particulates (UFPs) are able to pass deep into respiratory pathways and translocate past the alveolar–epithelial barrier, from the lungs to other organs via the blood [20].

Studies have reported that PM-exposed lungs release pro-inflammatory cytokines such as Interleukin-6 (IL-6) and C-reactive protein (CRP) into the blood stream [9]. As a result, they both lead to altering of cardiovascular functioning by increased blood viscosity and increased peripheral artery narrowing [21,22,23,24], which are associated with elevated BP [25,26,27]. PM could also affect BP by the triggered release of reactive oxygen species that upregulate BP signaling pathways [21,22].

We observed that PNC, but not BC or PM_2.5_, predicted short-term higher blood pressure after adjusting for temperature and individual-level confounding factors such as age, BMI, gender, smoking, and hypertension. Our data are consistent with an upregulating effect of PNC on the blood pressure of near-highway residing adults. PNC has been successfully used as a proxy for UFPs which are potentially more toxic than other PM size classes (*i.e.*, PM_10_, PM_2.5_) [17].

## 5. Limitations

Our analysis has numerous limitations. The air pollution was monitored at a central site, consistent with most studies of this sort; however, this study design likely leads to a degree of exposure misclassification. This is less of a problem for PM_2.5_, which is a regional pollutant. But for PNC, in particular, ambient levels vary considerably across small geographic areas [15]. Thus, it is likely that the association we observed with PNC was driven by the similarity of temporal changes in PNC levels at multiple locations due to shared traffic and meteorological patterns. There is also an error of plus or minus about 2 h in the assignment of the time of the BP measurements; however, this error is small relative to the 24-h period used in our analysis. There is additional error in the measurement of BP. We used an automatic machine, which reduces technician error. However, the stress of having BP taken affects some people. Using the average of two BP measures (right and left arm) can increase precision, but we cannot rule out that the first BP measure (on the right arm) was more likely to be affected by stress than the subsequent BP measures. Individual arm sensitivity analyses were consistent with our main analysis, suggesting this is likely not a serious problem. Lastly, residual confounding is still a concern, particularly with respect to controlling for confounding by temperature, which is an area in need of further study.

## 6. Conclusions

Our findings contribute to a modest, but growing literature assessing the association of PNC with BP. More research is needed to assess the potential for PNC to contribute to cardiovascular risk.

## Supplementary Files

Supplementary File 1
