# Supplementary material for: Association of PNC, BC, and PM2.5 Measured at a Central Monitoring Site with Blood Pressure in a Predominantly Near Highway Population
Source: Int J Environ Res Public Health. 2015 Mar 3;12(3):2765–80. doi: 10.3390/ijerph120302765 (PMC4377931; doi:10.3390/ijerph120302765)
Supplement: Supplementary File 1 [file ijerph-12-02765-s001.pdf]

Association of PNC, BC, and $PM_{2.5}$ Measured at a Central Monitoring Site with Blood Pressure in a Predominantly Near Highway Population

Table S1. Correlation matrix of 24-h PNC, PM2.5, BC pollutant, temperature, and quadratic-centering transformed temperature measure (*n* = 331 at both visits).

Exposure Variables	24-h PNC	24-h $PM_{2.5}$	24-h BC	24-h Temperature	Quadratic-Centering Transformed Temperature
**PNC**	1.0000				
**$PM_{2.5}$**	−0.0136	1.0000			
**BC**	0.3002	0.7951	1.0000		
**24-h Temperature**	−0.7547	0.0305	−0.1574	1.0000	
**Quadratic-Centering Transformed Temperature**	−0.1570	0.2627	0.1336	0.0252	1.0000

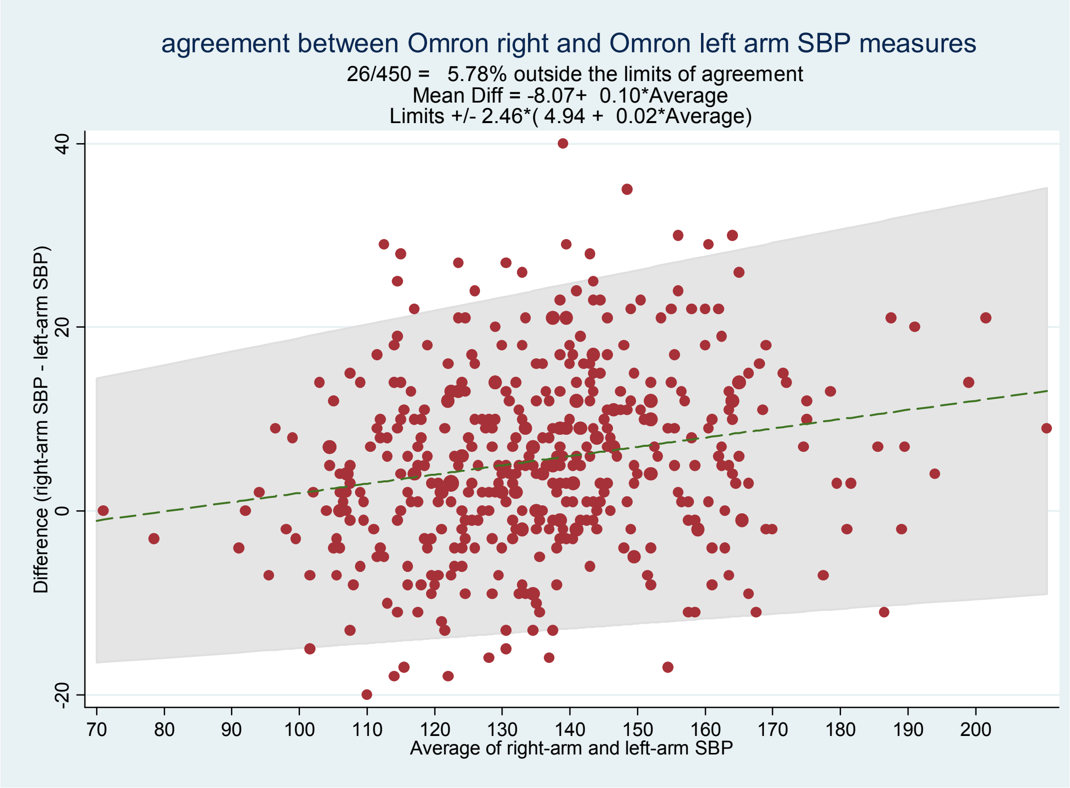

Figure S1. Bland-Altman Limits of Agreement analysis for the agreements between Omron right and left arm SBP measures.

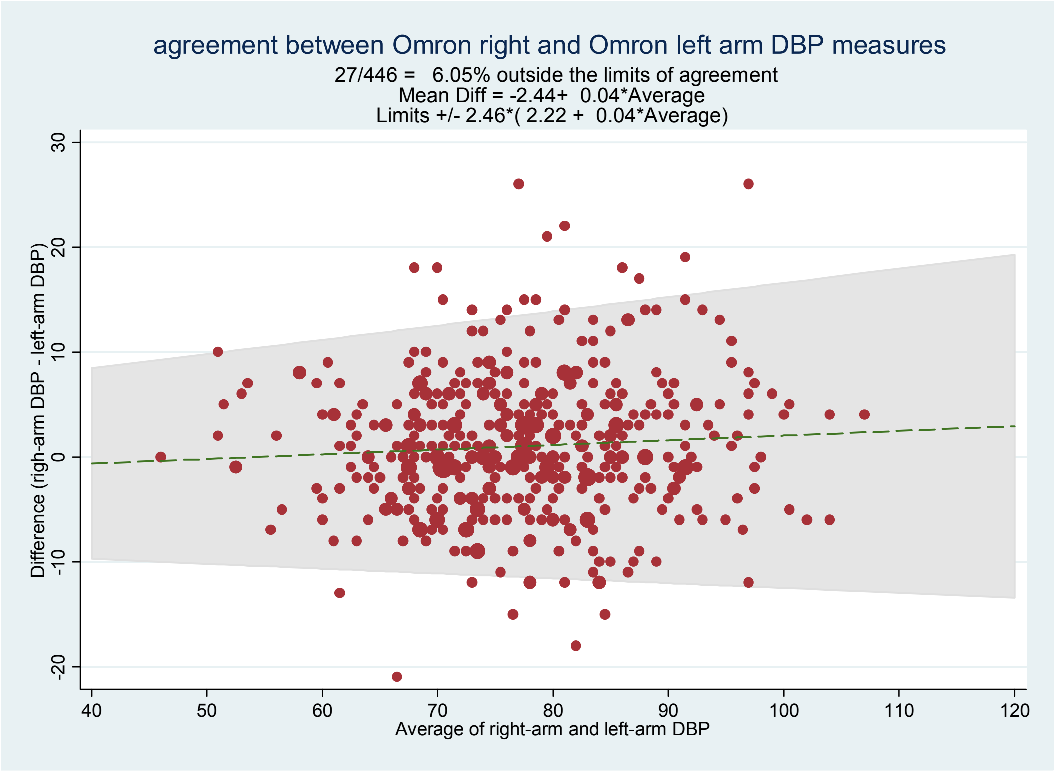

Figure S2. Bland-Altman Limits of Agreement analysis for the agreements between Omron right and left arm DBP measures.

Tables S2. Sensitivity analysis (number of observations = 302 in 190 people) using natural log of BP variables as the dependent variables.

**Air Pollutant**	**SBP**			**DBP**			**PP**		
	**R**	**Robust SE**	***p***	**R**	**Robust SE**	***p***	**R**	**Robust SE**	***p***
24 h PNC (10,000 particles/cm^3)	0.018	0.013	0.18	0.028	0.028	0.05	0.0000004	0.004	0.85
24 h PM2.5 (ug/m3)	−0.0016	0.0012	0.18	−0.0021	0.0015	0.15	−0.001	0.0021	0.62
24 h BC (μg/m^3)	−0.01	0.02	0.51	-0.02	0.02	0.37	−0.001	0.03	0.97

Notes: Covariates in the model: Temperature (quadratic term of the centering temperature variable), random/ convenience sample, highway proximity, age, gender, race, obesity status, hypertension medication use, smoking status, education status, seasonal variation (sin and cos), weekday or weekends, and clinic date. Beta coefficient (R) for each pollutant can be interpreted as % change per unit increase in the pollutant concentration as indicated in the table. * $p < 0.05$; ** $p < 0.01$.

Tables S3. Assessment of potential confounding by temperature in our PNC, PM2.5, or BC models.

Air Pollutant	Model	With Temperature Covariate		Without Temperature Covariate		% Change in R
		R	*p*	R	*p*	
24 h PNC (10,000 particles/cm^3)	SBP	2.20	0.226	2.24	0.213	2%
	DBP	2.41	0.030 *	2.32	0.038 *	−4%
	PP	−0.158	0.906	0.001	0.994	−94%
24 h PM2.5 (ug/m3)	SBP	−0.211	0.195	−0.315	0.039 *	49%
	DBP	−0.143	0.213	−0.172	0.109	21%
	PP	−0.063	0.583	−0.138	0.206	121%
24 h BC (μg/m^3)	SBP	−1.333	0.584	−2.364	0.321	−197%
	DBP	−1.257	0.458	−1.594	0.342	27%
	PP	−0.033	0.985	−0.745	0.668	2160%

Notes: Other covariates in the model: random/convenience sample, highway proximity, age, gender, race, obesity status, hypertension medication use, smoking status, education status, seasonal variation (sin and cos), weekday or weekends, and clinic date. * $p < 0.05$.

Tables S4. Sensitivity analysis using different arm BP measurements (number of observations = 300 in 190 people).

**(a) First Right-arm PNC Model**									
	**SBP**			**DBP**			**PP**		
	**R**	**Robust SE**	***p***	**R**	**Robust SE**	***p***	**R**	**Robust SE**	***p***
24 h PNC (10,000 particles/cm^3)	3.20	2.15	0.14	2.45	1.34	0.07	−1.58	1.34	0.91
$PM_{2.5}$ (ug/m^3)	−0.30	0.22	0.17	−0.14	0.16	0.38	−0.10	0.12	0.40
BC (ug/m^3)	−6.51	2.43	0.10	−2.38	1.57	0.13	−2.88	1.52	0.60
**(b) Repeated Right Arm PNC Model**									
	**SBP**			**DBP**			**PP**		
	**R**	**Robust SE**	***p***	**R**	**Robust SE**	***p***	**R**	**Robust SE**	***p***
24 h PNC (10,000 particles/cm^3)	4.05	2.07	0.05	2.33	1.34	0.08	−0.16	1.34	0.91
24 h $PM_{2.5}$ (ug/m^3)	−0.28	0.22	0.19	−0.24	0.15	0.12	−0.10	0.12	0.40
24 h BC (ug/m^3)	−1.88	3.51	0.59	−2.57	2.29	0.26	−2.88	1.52	0.06

Table S4. *Cont.*

(c) Left-arm Only PNC Model									
	**SBP**			**DBP**			**PP**		
	**R**	**Robust SE**	***p***	**R**	**Robust SE**	***p***	**R**	**Robust SE**	***p***
24 h PNC (10,000 particles/cm^3)	1.23	1.81	0.50	2.26	1.21	0.06	1.00	1.36	0.46
24 h $PM_{2.5}$ (ug/m^3)	−0.08	0.16	0.63	−0.08	0.12	0.53	0.01	0.12	0.95
24 h BC (ug/m^3)	1.41	2.34	0.55	−0.09	1.77	0.96	1.71	2.02	0.40

Notes: Covariates in the model: Temperature (quadratic term of the centering temperature variable), random/ convenience sample, highway proximity, age, gender, race, obesity status, hypertension medication use, smoking status, education status, seasonal variation (sin and cos), weekday or weekends, and clinic date.

© 2015 by the authors; licensee MDPI, Basel, Switzerland. This article is an open access article distributed under the terms and conditions of the Creative Commons Attribution license (http://creativecommons.org/licenses/by/4.0/).
